# A Novel Prediction Method of Transfer-Assisted Action Oriented to Individual Differences for the Excretion Care Robot

**DOI:** 10.3390/s23249674

**Published:** 2023-12-07

**Authors:** Yina Wang, Wenjie Hao, Yanjun Yu, Junyou Yang, Guang Yang

**Affiliations:** 1 School of Electrical Engineering, Shenyang University of Technology, Shenyang 110870, China; wenjiehao@smail.sut.edu.cn (W.H.); yuyanjun@sinocores.com (Y.Y.); junyouyang@sut.edu.cn (J.Y.); 2 Department of Intelligent Mechanical Systems Engineering, Kochi University of Technology, Kami 7828502, Japan; yang.guang@kochi-tech.ac.jp

**Keywords:** transfer care, multi-head attention, human three-dimensional model, bidirectional long- and short-term memory, individual differences

## Abstract

The excretion care robot’s (ECR) accurate recognition of transfer-assisted actions is crucial during its usage. However, transfer action recognition is a challenging task, especially since the differentiation of actions seriously affects its recognition speed, robustness, and generalization ability. We propose a novel approach for transfer action recognition assisted by a bidirectional long- and short-term memory (Bi-LSTM) network combined with a multi-head attention mechanism. Firstly, we utilize posture sensors to detect human movements and establish a lightweight three-dimensional (3D) model of the lower limbs. In particular, we adopt a discrete extended Kalman filter (DEKF) to improve the accuracy and foresight of pose solving. Then, we construct an action prediction model that incorporates a fused Bi-LSTM with Multi-head attention (MHA Bi-LSTM). The MHA extracts key information related to differentiated movements from different dimensions and assigns varying weights. Utilizing the Bi-LSTM network effectively combines past and future information to enhance the prediction results of differentiated actions. Finally, comparisons were made by three subjects in the proposed method and with two other time series based neural network models. The reliability of the MHA Bi-LSTM method was verified. These experimental results show that the introduced MHA Bi-LSTM model has a higher accuracy in predicting posture sensor-based excretory care actions. Our method provides a promising approach for handling transfer-assisted action individual differentiation in excretion care tasks.

## 1. Introduction and Related Works

In this aging society, the number of people with disabilities due to illness or accidents is increasing [1]. For those with severe disabilities, who are unable to stand and walk, daily toilet care is a complex and heavy task for their caregivers [2]. Particularly, the transfer task which is particularly important for toilet care is very burdensome and needs special body mechanics and the usage of proper techniques. Repeated lifting actions over a long period will harm the caregiver’s waist and reduce his or her work efficiency. Therefore, following appropriate techniques and utilizing excretion care robots (ECR) can make the transfer task more manageable and reduce the potential for injuries [3]. In the author’s lab, we developed an excretion care robot in our previous study [4]. To complete the transfer-assist task, the excretion care robot’s accurate recognition of transfer-assisted actions is crucial during its usage. Therefore, a highly reliable estimation of human posture and an accurate prediction of human movement is highly described.

Currently, research on predicting individualized movements for transferring and caring for the mobility-impaired is still in its early stages. However, various methods have been proposed for general motion prediction. Examples include methods based on vision, wearable sensors, and electromyography. However, specific scenarios like toilet care transfer also pose unique challenges. In addition to considering sensor applicability, predictive models must address issues of accuracy, real-time performance, and generalization capabilities in this context. The utilization of 3D human body models to capture motion characteristics is considered an effective approach, but existing models are computationally intensive and exhibit complexities in their expression, leading to a lag in reconstructing human motion. Furthermore, studies often focus solely on movements within the sagittal plane, with limited consideration for lateral swaying. Given that both predictive models and 3D human body model data are derived from sensors, ensuring high reliability, minimal error, and low drift in the data is crucial for this research.

Many methods exist for predicting human body movements. In terms of visual sensors [5], human body movements can be predicted by modeling the spatial information of the body’s skeletal points. Common models used for predicting human actions include convolutional neural network (CNN) models [6] and prediction models based on graph convolutional networks (GCN) [7]. Study [8] uses a multi-path convolutional network to learn the movement trajectory features of each joint in the human body. However, it is difficult for graph neural networks to fully model the correlations between one joint and other joints in the human body since they model the dynamic information of the human body based on nodes. In addition, during activities such as using the bathroom or getting on and off transportation, visual sensors may be affected by environmental factors such as lighting, as well as privacy concerns. In wearable sensor-based human action prediction [9], conventional methods typically rely on Markov assumptions, smoothness, or low dimensionality to simulate real movements and provide predictions. A study [10] proposed an ordered Markov chain to recognize and predict human action behaviors a few seconds earlier. However, the methods mentioned above are almost discrete, whereas human motion is continuous and nonlinear. Establishing a mapping model between joint angles and human movements based on wearable sensors is a feasible approach to addressing human motion prediction. This involves building a human model [11] to obtain the trajectory of human motions. Study [12] constructed an upper limb equivalent model based on the human anatomical structure and motion biomechanics model and evaluated and improved the trajectory planning of exoskeletons based on this model. However, current research in human modeling often simplifies computations by considering only movements in the sagittal plane [13,14]. Due to lower limb impairments, individuals with mobility difficulties may require assistance from others, causing their bodies to lean towards one side. In such cases, it is necessary to consider movements in the coronal plane when analyzing human motion trajectories. Furthermore, in three-dimensional modeling, there are challenges such as complex expressions and difficulties in reconstructing human motion [15]. The complexity of the model and its expressions can result in a certain degree of lag in the model’s predictions. However, wearable sensors in practical applications are susceptible to various environmental factors. For example, accelerometers and gyroscopes are subject to measurement noise, biases, drift, and other influencing factors during their usage [16]. The extended Kalman filter (EKF) [17] is a suitable choice for handling nonlinear motion which has excellent noise-handling capabilities.

The transfer-assisted action of the human body during transferring is influenced by individual differences among caregivers, transfer times, and environmental variations. The differences in transfer actions often lead to variations in movement trajectories. This complexity makes it challenging to predict the movement trajectory of the hips during toileting for a caregiver. Many researchers have employed artificial neural networks (ANN) [18], support vector machines (SVM) [19], and Gaussian mixture prediction models [20] for this purpose. However, these models often require high computational costs. Currently, the LSTM neural network has demonstrated outstanding advantages in its predictions compared to traditional prediction models. Its strength lies in its ability to learn long-term dependencies [21]. It is an improved version of a recurrent neural network, effectively addressing the issues of gradient explosion and gradient vanishing. The LSTM algorithm has been widely applied in time series prediction problems [22,23]. In study [24], LSTM was used to predict multiple time frames of gait, and the predicted results were highly correlated with the measured trajectories. As the application of LSTM deepens, various structural variants derived from it and algorithms that leverage the good performance of LSTM are still being explored. The well-known basic structure of LSTM is a unidirectional network, which only considers information from previous frames to learn future states, leading to inaccurate predictions. Another variant of LSTM called bidirectional long short-term memory (Bi-LSTM) [25,26,27] can process input time series in both forward and backward directions. When making predictions, it considers both past and future information, capturing a more comprehensive context and resulting in coherent predictions that are closer to the true values. In reference [28], a motion trajectory tracking system based on inertial measurement units (IMUs), residual neural networks, and Bi-LSTM is proposed. This system accurately predicts daily life activities. Besides considering the model’s prediction accuracy, we also need to evaluate the generalization performance of the model to ensure that it meets the requirements. To improve the generalization of the model and effectively learn time series features, many researchers have started to introduce attention mechanisms (AM) [29] and their variant structures to enhance the model’s ability to generalize. During the process of assisting individuals in toileting and transferring, the movement trajectory of the individual’s hips is influenced by individual differences, temporal variations, and environmental factors. This necessitates the model to have a high level of generalization to accommodate these variabilities. In response to the aforementioned requirements and existing challenges, we present a novel continuous dynamic trajectory prediction approach based on the MHA Bi-LSTM neural network. The primary objective of this research is to address the issue of predicting motion trajectories characterized by distinctive properties. The MHA exhibits the capability of simultaneously considering multiple attention weights to extract crucial information from different dimensions. Moreover, it can automatically select and focus on the most relevant information. This ability enables the model to better capture important features within diverse motion patterns. The Bi-LSTM neural network combines the flow of information in both forward and backward directions, allowing for the effective utilization of past and future contextual information. This bidirectional modeling capability enables the model to better comprehend the temporal dependencies and dynamic variations within motion trajectories. In summary, the combination of the MHA and Bi-LSTM neural networks allows for the comprehensive utilization of critical information from different dimensions and the effective modeling of temporal dependencies and dynamic variations. This integration improves the accuracy and generalization of dynamic trajectory prediction.

The contribution of this paper is as follows:

(1) Develop a 3D model considering the lateral displacement in human motion. To address the issue of lateral displacement in individuals with lower limb weakness, establish the relationship between the angle of coronal plane displacement and lateral displacement using the cosine theorem. Based on geometric relationships, establish a 3D model for the lower limbs to obtain the continuous and dynamic trajectories of human transfer-assisted actions.
(2) To address the issue of differentiated transfer-assisted actions, a prediction method based on MHA Bi-LSTM is proposed. By incorporating MHA, key information related to differentiated actions can be extracted from different dimensions and given input weights. The Bi-LSTM network is utilized to effectively consider both past and future information, resulting in more accurate prediction outcomes. This model not only demonstrates strong generalization capabilities but also accurately forecasts multiple frames of motion.

The rest of this paper is presented as follows: in Section 2, the hardware architecture of the system and the multi-sensor data acquisition system are presented; Section 3 describes the methods used in this paper including the DEKF, the construction of a 3D model of human the human body, and the proposed MHA Bi-LSTM for transfer-assisted actions with differentiated characteristics; Section 4 presents a discussion of the extensive experimental details of this paper; and Section 5 gives our experimental conclusions.

## 2. Hardware Introduction

### 2.1. Structure of the ECR

**Figure 1:**
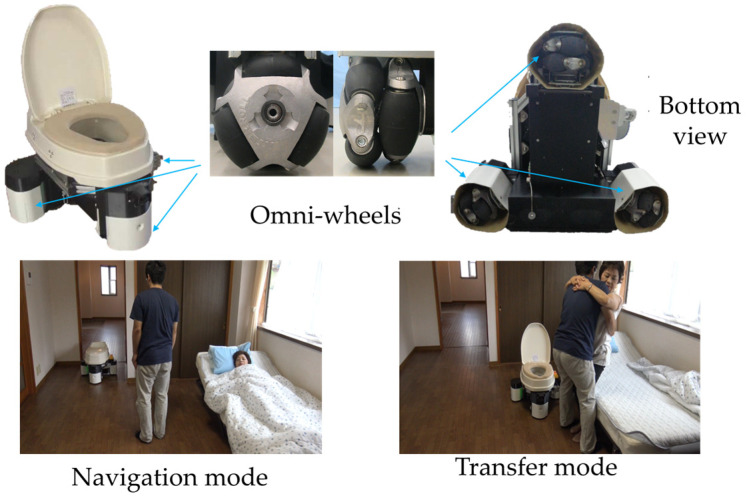
ECR and its working modes.

The structure of our developed ECR is shown in Figure 1. Three omnidirectional wheels are mounted on the bottom of the robot to enable the omnidirectional movement of the robot. As shown in Figure 1, the robot has two working modes. The navigation mode denotes that the robot can autonomously move from a standby position to the user’s side. In contrast, the transfer mode denotes that the robot can cooperate with caregivers in completing transfer tasks. In this paper, we will focus on the transfer mode to develop the transfer actions prediction for the robot.

### 2.2. Data Detection System

**Figure 2:**
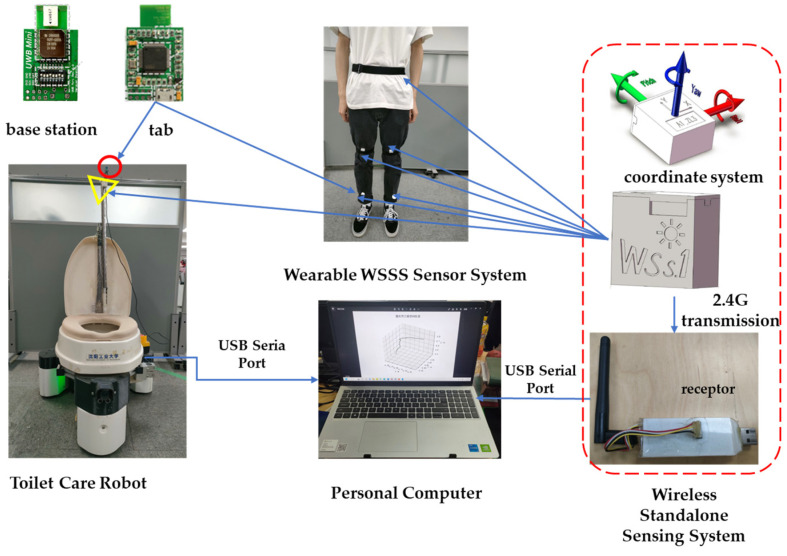
Data acquisition systems.

To cooperate with the caregiver in the transfer task, the robot needs to be able to predict the user’s position and posture in real-time. We built the experimental field area in the lab based on the practical use of the UWB localization system, which is 6 m × 8 m. The experimental setups we use are the Wireless Standalone Sensing System (WSSS) module, the Ultra-Wideband (UWB) module, and the ECR. The WSSS is installed on the right and left calves, the right and left thighs, and the waist to measure the joint angles and angular velocities of the human body. The ECR also needs to be installed with the WSSS to determine the posture of the toilet. A UWB tag is installed at the right ankle position of the body for determining the position of the body and a UWB tag is also installed on the ECR for localization. The data acquisition system is shown in Figure 2, where the WSSS uses a high-speed 2.4G wireless data communication protocol and a wireless receiver module connected to a computer to receive data. This wireless transmission system provides quaternions and Euler angles (Yaw, Pitch, Roll) in real time with an angular resolution of 0.01 degrees. The UWB positioning system consists of five UWB modules, three base stations, and two tags. We realize the two-dimensional location of the tags by measuring the time of flight between two asynchronous transceivers.

## 3. Methodology

### 3.1. Human Body 3D Modeling

**Figure 3:**
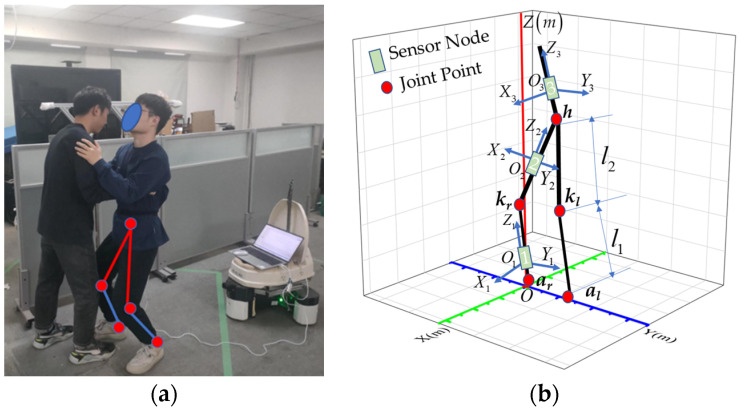
3D modeling of the human lower limbs. (**a**) Assistance in sitting transformation. (**b**) Five-link model. Where *O* (*X*, *Y*, *Z*) denotes the world coordinate, *a_r_* (*x*_1_, *y*_1_, *z*_1_) is the position of the right ankle joint, *k_r_* (*x*_2_, *y*_2_, *z*_2_) denotes the position of the right knee joint, *h* (*x*_3_, *y*_3_, *z*_3_) denotes the position of the body hip, *k_l_* (*x*_4_, *y*_4_, *z*_4_) denotes the frame of the left knee joint, and *a_l_* (*x*_5_, *y*_5_, *z*_5_) denotes the position of the left ankle joint. The coordinate systems of sensor 1 are *O*_1_ (*X*_1_, *Y*_1_, *Z*_1_), the coordinate systems of sensor 2 are *O*_2_ (*X*_2_, *Y*_2_, *Z*_2_), and the coordinate systems of sensor 3 are *O*_3_ (*X*_3_, *Y*_3_, *Z*_3_). *l*_1_ and *l*_2_ are the lengths of the right calf and right thigh, respectively.

Since the transfer assist action mainly focuses on the waist and lower limb movement state, the 3D model of the human body can be generalized to the forms shown in Figure 3. The human body in Figure 3a is simplified into five joints and five links, as shown in Figure 3b.

**Figure 4:**
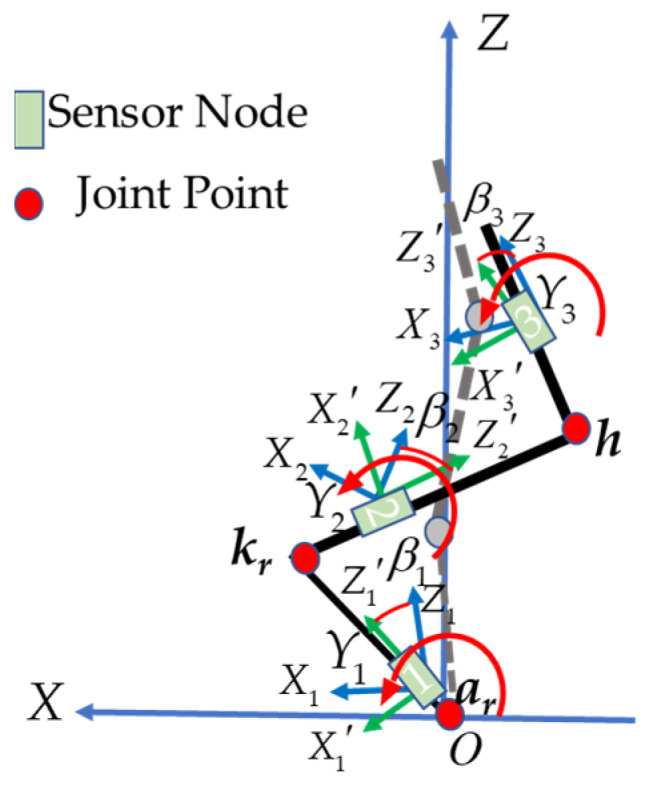
Sagittal plane motion schematic of the human body. Angles *β*_1_, *β*_2_, and *β*_3_ denote the rotations of sensor 1, sensor 2, and sensor 3 around the *y*-axis, respectively. The positional changes of the knee joint and hip joint in the sagittal plane can be solved based on geometric relationships and human body parameters.

Firstly, we consider the human body action of the three-link model in the sagittal plane based on Figure 4.

**Figure 5:**
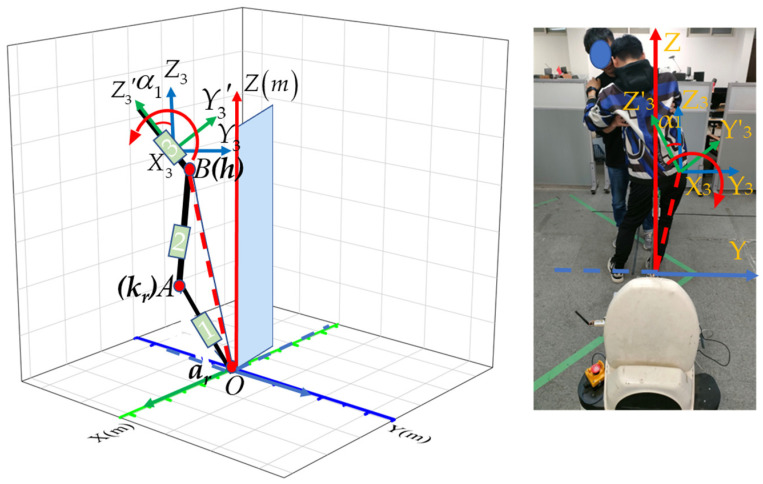
Schematic diagram of rightward sway during sitting down movements.

Furthermore, during the process of sitting down, individual variations in lower limb weakness and temporal differences can result in deviations from vertical motion. This can lead to a deviation from vertical motion when the human body sits down. Therefore, it is necessary to analyze the motion in the coronal plane when establishing a model. Figure 5 provides detailed information on the action in the coronal plane when the human body sits down.

The action of the human body in the coronal plane reflects the lateral swaying during sitting down movements, where *α*_1_ represents the angle of rotation around the *X*-axis, which corresponds to the lateral offset of the body. We establish the relationship between the lateral offset distance and *α*_1_ using the cosine theorem.

Motion in the coronal plane is solved using the cosine theorem and the offset angle to find the left–right offset distance of the body. The body’s left–right offset distance can be obtained through Equation (1).

(1) $$\left\{\begin{array}{l} ∠A=\beta_{1}+\beta_{2}\\ OB=l_{1}^{2}+l_{2}^{2}-2l_{1}l_{2}\cos OAB\\ y_{3}=OB\times \sin\alpha_{1} \end{array}\right.$$

In this case, *OB* is the distance from the lower limb buttocks to the right ankle of the human body, and *α*_1_ is the angle at which the hips are offset to the left or right.

Therefore, the 3D movement of the hip position is shown in Equation (2):

(2) $$\left\{\begin{array}{l} x_{3}=l_{2}\cos\beta_{2}-l_{1}\cos\beta_{1}\\ y_{3}=OB\times \sin\alpha_{1}\\ z_{3}=l_{1}\sin\beta_{1}+l_{2}\sin\beta_{2} \end{array}\right.$$

### 3.2. Discrete Extended Kalman Filter

We utilize a discrete extended Kalman filter analysis to process data from lower limb sensors on the human body and obtain the motion trajectory of the human body’s hips in a 3D kinematic model.

Taking the lower limb movement of the human body as an example, the corresponding nonlinear system can be expressed as Equation (3):

(3) $$\left\{\begin{array}{l} X_{k+1}=\Phi_{k+1,k}X_{k}+W_{k}\\ Z_{k+1}=H_{k+1}X_{k+1}+V_{k+1} \end{array}\right.$$

where *X_k_*_+1_ indicates the estimated value at the current moment, *X_k_* denotes the best estimate at the previous moment, *W_k_*_+1_ and *V_k_*_+1_ are the white noise where both system noise and measurement noise have zero means and are uncorrelated, *Z_k_*_+1_ represents the measured value, Φ*_k_*_+1,*k*_, and *W_k_* represent the Jacobian matrix obtained by taking the partial derivatives of the nonlinear system function at time *k*, and *H_k_*_+1_ and *V_k_*_+1_ represent the Jacobian matrix obtained by taking the partial derivatives of the nonlinear measurement function at time *k* + 1. *H_k_*_+1_ is the measurement equation of the system. The Φ*_k_*_+1,* k*_ are shown in Equation (4).

(4) $$\Phi_{k+1,k}={\left[\begin{array}{ccc} 1+\cos\alpha_{k}\tan\beta_{k}\omega_{yk+1}T-\sin\alpha_{k}\tan\beta_{k}\omega_{zk+1}T & \frac{\sin\alpha_{k}}{\cos\beta_{k}^{2}}\omega_{yk+1}T+\frac{\cos\alpha_{k}}{\cos\beta_{k}^{2}}\omega_{zk+1}T & 0\\ -\sin\alpha_{k}\omega_{yk+1}T-\cos\alpha_{k}\omega_{zk+1}T & 1 & 0\\ \frac{\cos\alpha_{k}}{\cos\beta_{k}}\omega_{yk+1}T-\frac{\sin\alpha_{k}}{\cos\beta_{k}}\omega_{zk+1}T & \frac{\sin\alpha_{k}\sin\beta_{k}}{\cos\beta_{k}^{2}}\omega_{yk+1}T+\frac{\cos\alpha_{k}\sin\beta_{k}}{\cos\beta_{k}^{2}}\omega_{zk+1}T & 0 \end{array}\right]}_{3\times 3}$$

where the Euler angles = [*α_i_*, *β_i_*, *γ_i_*] and angular velocities = [*ω_xi_*, *ω_yi_*, *ω_zi_*] of Sensor 1, Sensor 2, and Sensor 3 were individually measured, and *T* is the time step, *i* = 1, 2, 3.

The implementation process of the DEKF consists of two main parts: the prediction and the update.

(1) The prediction step utilizes the estimate from the previous time step *k*, to generate the estimate for the current time step *k* + 1. The prediction phase is completed in two steps, as shown in Equation (5):

(5) $$\left\{\begin{array}{l} {\hat{X}}_{k+1/k}=\Phi_{k+1,k}{\hat{X}}_{k}\\ P_{k+1/k}=\Phi_{k+1,k}P_{k}\Phi_{k+1,k}^{T}+Q \end{array}\right.$$

where ${\hat{X}}_{k+1/k}$ is the state variable at the moment *k* + 1 based on the prediction at moment *K*, *P_k_* is the optimal estimated covariance matrix at the previous moment, *P_k+_*_1/*k*_ is the a priori estimate of the state vector covariance at moment *k* + 1, and *Q* is the variance matrix of the system noise series. The initial value is to be given at start-up *X*_0_ and *P*_0_. *X*_0_ = [*α*_0_, *β*_0_, *γ*_0_].

(2) The update is done in three steps as shown in Equation (6):

(6) $$\left\{\begin{array}{l} K_{k+1}=P_{k+1/k}H_{k+1}{\left(H_{k+1}P_{k+1/k}H_{k+1}^{T}+R_{k+1}\right)}^{-1}\\ {\hat{X}}_{k+1}={\hat{X}}_{k+1/k}+K_{k+1}\left(Z_{k+1}-H_{k+1}{\hat{X}}_{k+1/k}\right)\\ P_{k+1}=\left(I-K_{k+1}H_{k+1}\right)P_{k+1/k} \end{array}\right.$$

where *K_k+_*_1_ is the weighted array of residuals, called the Kalman filter gain array, and *R_k+_*_1_ is the measurement noise variance matrix. ${\hat{X}}_{k+1/k}$ is the state vector at moment *k* + 1, ${\hat{X}}_{k+1/k}$ is the a priori estimate at moment *k* + 1, and *Z_k_* is the sensor measurement value and is the linearized measurement matrix.

By iteratively performing the prediction and update steps, the DEKF continuously refines its state estimate based on the available measurements, providing an improved estimation of the system’s underlying dynamics.

### 3.3. Prediction Model of Transfer Assist Action

To achieve timely assistance for different individuals, the robot must have the function of forward-looking prediction for the transfer assist action. Therefore, we propose a 3D path prediction model for the human sitting process based on the MHA Bi-LSTM neural network. By predicting the future trajectory of human sitting, the robot can arrive at the toilet location in advance and assist caregivers promptly.

**Figure 6:**
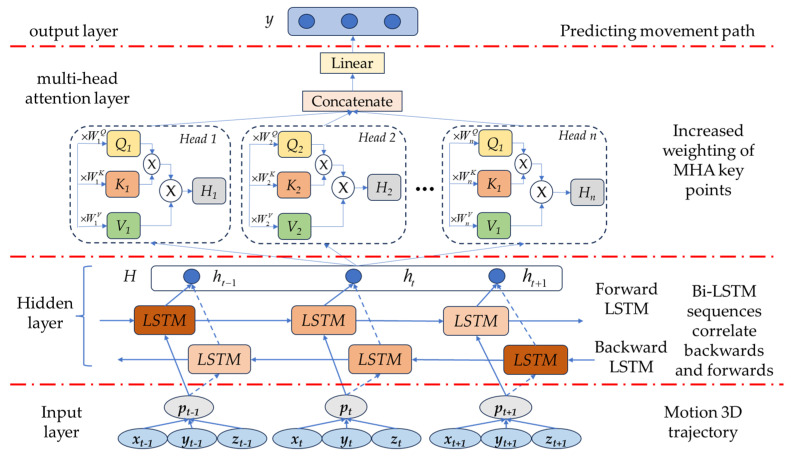
MHA Bi-LSTM prediction structure.

The architecture of the MHA Bi-LSTM model, as depicted in Figure 6, consists of four components: an input layer, a single layer of bidirectional LSTM encoders, a multi-head attention layer, and an output layer. The input layer receives the hip motion trajectory *p* = [*p*1, *p*2, *p*3, …] from the Section 3.2, where *P_i_* = [*x_i_*, *y_i_*, *z_i_*] represents the three-dimensional coordinates at each time step (*i* = 1, 2, 3, …). The bidirectional LSTM encoder component encodes the input three-dimensional motion trajectory separately. We incorporate a multi-head attention mechanism between the hidden layer of the bidirectional LSTM and the fully connected layer for decoding. The decoder’s output is encoded into a vector sequence containing multiple subsets. The multi-head attention layer selectively picks a subset from the vector window at each decoding step to focus on more important information within the time series. This approach avoids directly encoding all input sequences into a fixed-length vector using the encoder. Finally, a linear activation function transforms the decoder’s output into predicted results.

#### 3.3.1. Bidirectional Long- and Short-Term Memory Neural Network Model

**Figure 7:**
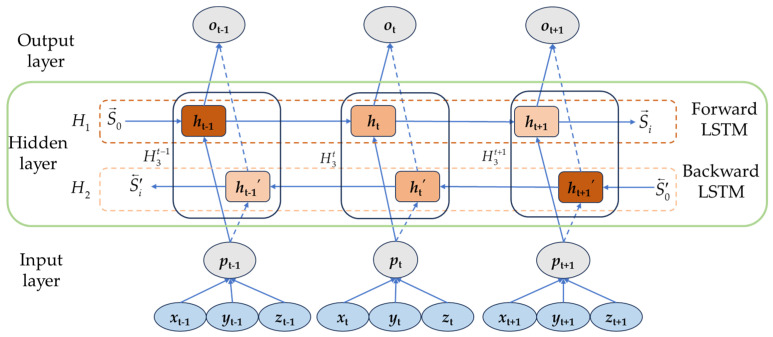
Bi-LSTM module.

The Bi-LSTM is a crucial component of the decoder in the MHA Bi-LSTM model. It performs forward and backward learning on the same time sequence through two layers of LSTM networks. The ability for bidirectional modeling in the Bi-LSTM allows for a better understanding of the temporal dependencies and dynamic variations in motion trajectories. The memory units and forget gates in the Bi-LSTM enable the model to flexibly remember and forget information from different time steps. This capability allows the model to better adapt to different motion patterns and exhibit a stronger generalization ability. The Bi-LSTM module details are shown in Figure 7.

First, we feed the 3D path time series into the forward LSTM network layer, and the first set of forward hidden states $H_{1}=\left\{\left.{\vec{S}}_{1},{\vec{S}}_{2},\ldots ,{\vec{S}}_{i}\right\}\right.$, is computed as:

(7) $${\vec{S}}_{i}=f\left(\vec{U}\cdot x_{i}+\vec{W}\cdot {\vec{S}}_{i-1}+\vec{b}\right)$$

where ${\vec{S}}_{i}$ is the state of the forward hidden layer at moment *t*, *f* is the activation function, and $\vec{W}$ is the weight of the forward hidden layer at moment *t*. $\vec{b}$ is the bias of the forward hidden layer at moment *t*. *x_i_* is the sample input at moment *t*.

Secondly, the input samples to the inverted backward LSTM network layer are used to compute the second set of hidden layer states $H_{2}=\left\{\left.{\overset{\leftarrow }{S}}_{i},{\overset{\leftarrow }{S}}_{i-1},\ldots ,{\overset{\leftarrow }{S}}_{1}\right\}\right.$, computed as:

(8) $${\overset{\leftarrow }{S}}_{i}=f\left(\overset{\leftarrow }{U}\cdot x_{i}+\overset{\leftarrow }{W}\cdot {\overset{\leftarrow }{S}}_{i+1}+\overset{\leftarrow }{b}\right)$$

where ${\overset{\leftarrow }{S}}_{i}$ is the reverse hidden layer state at time *t*, *f* is the activation function, $\overset{\leftarrow }{W}$ is the weight of the reverse hidden layer at moment *t*, $\overset{\leftarrow }{b}$ is the bias of the reverse hidden layer at moment *t*, and *x_i_*, and is the sample input at time *t*.

Finally, in the network structure, both the forward and backward transmission layers are connected to the output layer, and the two sets of computed hidden layer states are spliced together to obtain $H_{3}=\left\{\left.\left[{\vec{S}}_{1},{\overset{\leftarrow }{S}}_{1}\right],\left[{\vec{S}}_{2},{\overset{\leftarrow }{S}}_{2}\right],\ldots ,\left[{\vec{S}}_{i},{\overset{\leftarrow }{S}}_{i}\right]\right\}\right.$, with the final output computed as:

(9) $$o_{i}=g\left(V\cdot \left[{\vec{S}}_{i},{\overset{\leftarrow }{S}}_{i}\right]+c\right)$$

where *o_i_* is the output at time *t*, *g* is the activation function, *V* is the weight of the output layer, and *c* is the output layer bias.

#### 3.3.2. Multi-Head Attention Mechanism

We place the MHA mechanism between the hidden layer and the fully connected layer of the Bi-LSTM. The hidden layer states obtained from the Bi-LSTM are used as inputs to the MHA mechanism. Firstly, the input data sequence is mapped to the information of concern using a query matrix (*Q*). A key matrix is then used to compute the similarity between different queries, and a value matrix contains the corresponding information for each position. The similarity between the query (*Q*) and the key (*K*) is used to calculate the attention weights for each position concerning the query. Typically, the similarity is computed using a dot product. The attention representation for each head is obtained by weighting and summing the values based on the attention weights. Finally, the attentional representations from all heads are concatenated or merged to obtain the final multi-head attentional representation. The process of realization is as follows:

(1) The output results in *H* from the Bi-LSTM are transformed through linear transformations to obtain the query (*Q*), key (*K*), and value (*V*):

(10) $$\left\{\begin{array}{l} Q=H*W_{q}\\ K=H*W_{k}\\ V=H*W_{v} \end{array}\right.$$

where *H* represents the hidden state of the Bi-LSTM module, and are the weight matrices of *Q*, *K*, and *V*, respectively.

(2) By transforming the *Q*, *K*, and *V* from Formula (10) into three input matrices with dimensions *d_k_* through different mapping operations, the attention output matrix is generated.

(11) $$Att(Q,K,V)=soft\max\left(\frac{QK^{T}}{\sqrt{d_{k}}}\right)V$$

where *d_k_* represents the feature dimension of the keys, used for weight scaling, Softmax is used to normalize the attention weights.

(3) The multi-head attention mechanism divides the time series into h different subspaces. Each head performs attention calculation on its corresponding subspace, thereby enhancing the expressive power. The results of each head are then concatenated to form multiple heads. Finally, through a linear transformation, the final result of *head_i_* is obtained.

(12) $$head_{i}=Att\left(QW_{i}^{Q},KW_{i}^{K},VW_{i}^{V}\right)$$

where $W_{i}^{Q},W_{i}^{K}\;\mathrm{and}\;W_{i}^{V}$ represent the weight matrices for *Q*, *K*, and *V*, respectively, and *head_i_* represents the *i*-th module of multiple attention heads.

(13) $$Multi\left(Q,K,V\right)=Concat\left(head_{1},\ldots ,head_{n}\right)W^{o}$$

where *W*${}^{o}$ is the weight matrix used for linear transformation, *Concat* represents the concatenation operation, and *Multi* (*Q*, *K*, *V*) is the output result.

(4) Finally, the final output of MHA Bi-LSTM is the time series obtained by applying a linear activation function to the output of MHA.

(14) $$y=Linear\left(Multi\left(Q,K,V\right)\right)$$

where *Linear* is the activation function, and *y* represents the final prediction result.

## 4. Experiment

The experiments in this section are divided into two parts: a 3D human model experiment based on DEKF to acquire human lower limb motion trajectories, and a human hip motion trajectory prediction experiment based on MHA Bi-LSTM. The experiments were conducted in a laboratory setting with three subjects who provided informed consent and complied with ethical regulations. The average age, height, and weight of the three subjects were 23 years, 175 cm, and 62 kg, respectively. When doing the transfer maneuver for assisted toileting, we ask that it be the same caregiver assisting. To avoid a distortion of the caregiver-assisted movements, each movement will be separated by a period. Each subject was asked to perform three different maneuvers: sitting normally (meaning a small left–right shift), shifting to the left, and shifting to the right. The collected movement data were then constructed into a separate dataset to be used as a training model.

The experimental environment was as follows: CPU: 11th Gen Intel(R) Core (TM) i5-1135G7 2.42 GHz, Memory: 8GB, Development Environment: Win 11, Programming Language: Python, and Software Used: Visual Studio 2017.

### 4.1. Validation of the 3D Model

To verify the validity of the model, we assisted the human body to do transfer-assisted actions from standing to sitting, including a downward sitting movement with no offset, a downward sitting movement with a rightward offset, and a downward sitting movement with a leftward offset. The measurement equation and the initial covariance matrix *P*_0_ of DEKF and the 3D model are given as shown in Equation (15).

(15) $$H_{k+1}={\left[\begin{array}{ccc} 1 & 0 & 0\\ 0 & 1 & 0\\ 0 & 0 & 1 \end{array}\right]}_{3\times 3},P_{0}={\left[\begin{array}{ccc} 10 & 0.3 & 0.3\\ 0.3 & 10 & 0.3\\ 0.3 & 0.3 & 10 \end{array}\right]}_{3\times 3}$$

The variance matrix of the system noise is *Q*_0_ = *Diag* (0.005, 0.005, 0.005), and the variance matrix of the measurement noise is *R_k_*_+1_ = *Diag* (5, 5, 5). In addition, in the 3D model, we set the length of the thigh as *l*_1_ = 0.5 m, and the length of the calf as *l*_2_ = 0.4 m.

**Figure 8:**
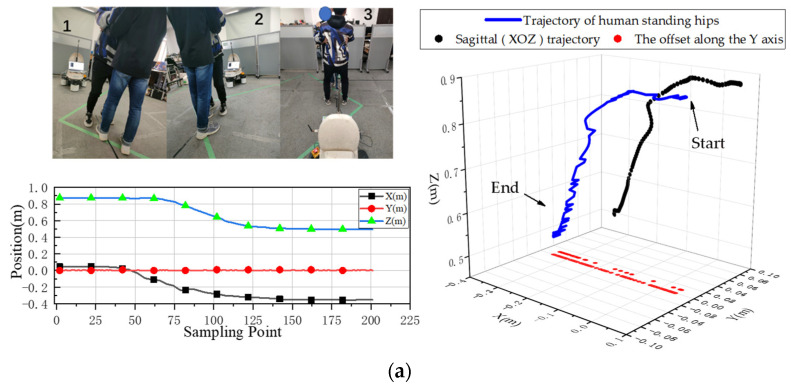

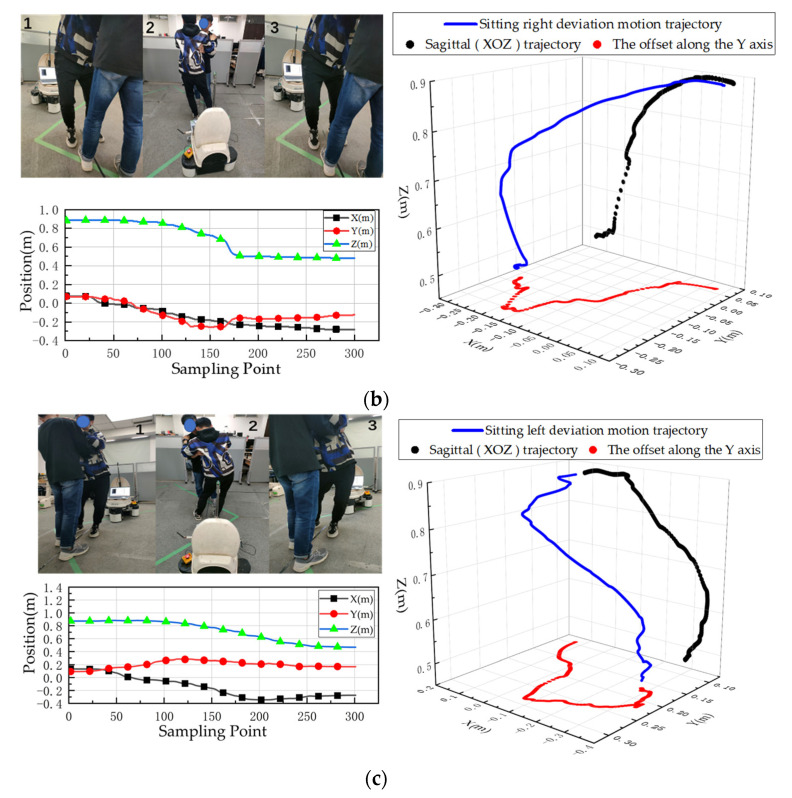
Consider three situations in which the human body sits down. (**a**) Down-sit without offset. (**b**) Down-sit right offset. (**c**) Down-sit left offset.

Figure 8a–c provides the experimental details of the three different sets of downward sitting motions, the trajectories of the hips during the downward sitting motions, and the positional changes of the hips in the *X*, *Y*, and *Z* directions during the downward sitting motions.

**Figure 9:**
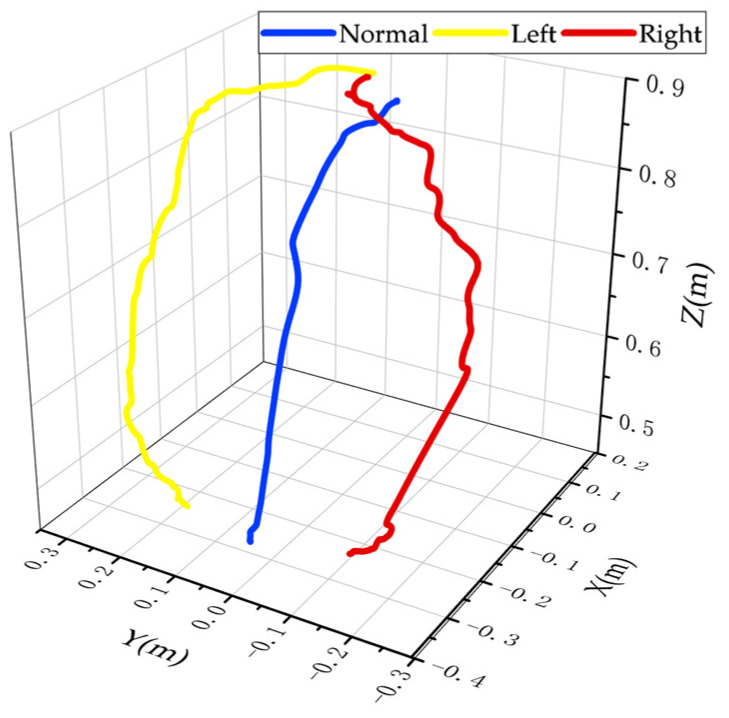
Comparison of three down-sit paths.

As shown in Figure 8, the path of the hip is given in the 3D path as a blue line, the path in the sagittal plane as a black line, and the offset in the *Y*-axis as a red line. The offset in the *Y*-axis direction can be derived from the XYZ position change diagram. A comparison of the three down-sit paths is shown in Figure 9.

**Figure 10:**
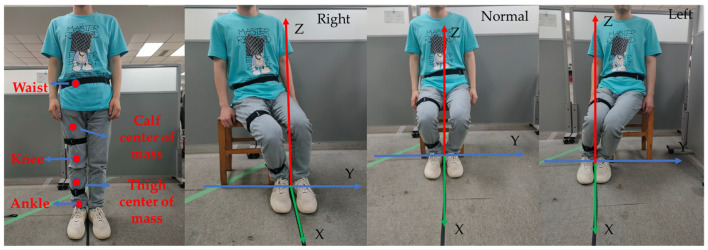
Multi-point calibration experiments.

To validate the validity and accuracy of the 3D model constructed in this study, we used a multi-point calibration measurement method, which utilizes pre-calibrated seated positions and allows the person to sit down in the calibrated position, during which a total of five dynamic positions are measured at the ankle, knee, hip, calf center of mass, and thigh center of mass of the lower limb, and are then compared to the calibrated position to validate the validity and accuracy of the model. The height of the bench was 0.45 m. A1 and A2 were used to represent the knee, B1 and B2 the hip, C1 and C2 the center of mass of the lower leg, and D1 and D2 the center of mass of the thigh. The experimental details of the multi-point calibration validation method are given in Figure 10, and Table 1 gives the measured and calibrated data for a variety of standing-to-sit points.

As can be seen from Table 1, we have calibrated five points in total, and as we have specified that there is no relative sliding between the foot and the ground in this study, only the four points of the knee, hip, calf center of mass, and thigh center of mass are considered for the accuracy of the model solution. Table 2 shows that the *Z*-axis for the knee, thigh center of mass, and hip are slightly higher than the calibrated values due to the influence of the measurement technique. The final error can be kept within ±4 cm.

### 4.2. Validation of Assist Action Prediction Model Considering Individual Differences

To construct the MHA Bi-LSTM prediction model, we used a grid search method to adjust the number of layers and nodes of the hidden layer of the Bi-LSTM network to find the optimal parameters. The best combination of parameters was found. The experimental parameters are set as follows: the learning rate is 0.0005, the number of nodes in the hidden layer is 128, the number of fully connected layers is 3, the number of neurons in the fully connected layer is 128, the number of training ephemera is 100, and the Batch size is 32. The discard rate of the discarded layer is 0.3. The number of parallel running attention mechanisms we set is 5, indicating that there are 5 heads. The CNN layer is used to extract the high dimensional features of the time series as inputs to the Bi-LSTM, whose optimal parameters are obtained using the grid search method. The CNN consists of a setup of a total of two layers, which are the convolutional layer and the maximum pooling layer. Where the convolutional layer’s filters = 64, kernel_size = (3, 3, 3), the activation = ‘relu’, padding = ‘same’, MaxPooling: pool_size = (2, 2, 2), strides = (2, 2, 2), and padding = ‘same’.

We have selected eight different individual shift multiplication action datasets, which include data from three individuals, and to reflect the variability of time, the dataset includes three different sampling times, because the shift multiplication time of different individuals in the real environment is also one of the key factors affecting the experiments. We also tested the prediction performance of the models on the test set for three different models with different shares of the test set and training set by comparing the prediction accuracy of the three algorithms with the MHA LSTM and MHA + CNN + Bi-LSTM.

**Figure 11:**
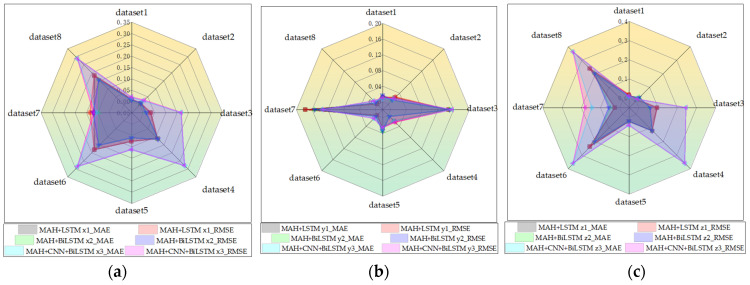
(**a**–**c**) Evaluation of the RMSE and MAE of three prediction models on eight data.

The comparison of RMSE and MAE for three different algorithms on different datasets for *X*, *Y*, and *Z* directions regarding the prediction results are given in Figure 11a–c, respectively, and visualized in radar plots. The prediction results of the test set are depicted in line graphs. The MAE of MHA + BiLSTM for the *X*-axis prediction is 0.155, which is smaller than the 0.182 MAE for MHA + CNN + BiLSTM and the 0.287 MAE for MHA + LSTM. The RMSE of MHA + BiLSTM for the *X*-axis prediction is 0.155, which is smaller than the 0.182 RMSE for MHA + CNN + BiLSTM and the 0.287 RMSE for MHA + LSTM. For the *Y*-axis analysis, the MAE predicted by MHA + BiLSTM for the *Y*-axis is 0.153, which is smaller than the 0.157 MAE for MHA + LSTM and the 0.177 MAE for MHA + CNN + BiLSTM. The MAE predicted by MHA + BiLSTM for the *Y*-axis is 0.153 less than the 0.157 MAE for MHA + LSTM and the 0.177 MAE for MHA + CNN + BiLSTM. In the *Z*-axis analysis, the MAE predicted by MHA + BiLSTM for the *Z*-axis is 0.2036 less than the 0.3621 MAE for MHA + LSTM and the 0.2384 MAE for MHA + CNN + BiLSTM. The MAE predicted by MHA + BiLSTM for the *Z*-axis is 0.2036 less than the 0.3622 MAE for MHA + LSTM and the 0.2384 MAE for MHA + CNN + BiLSTM.

**Figure 12:**
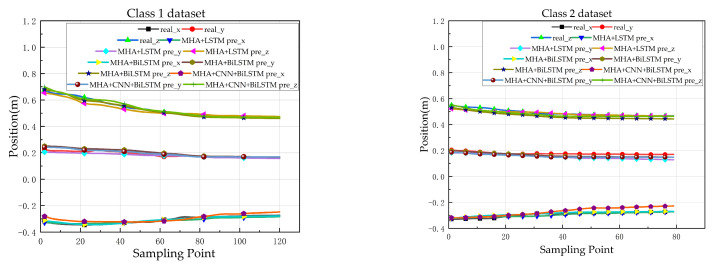

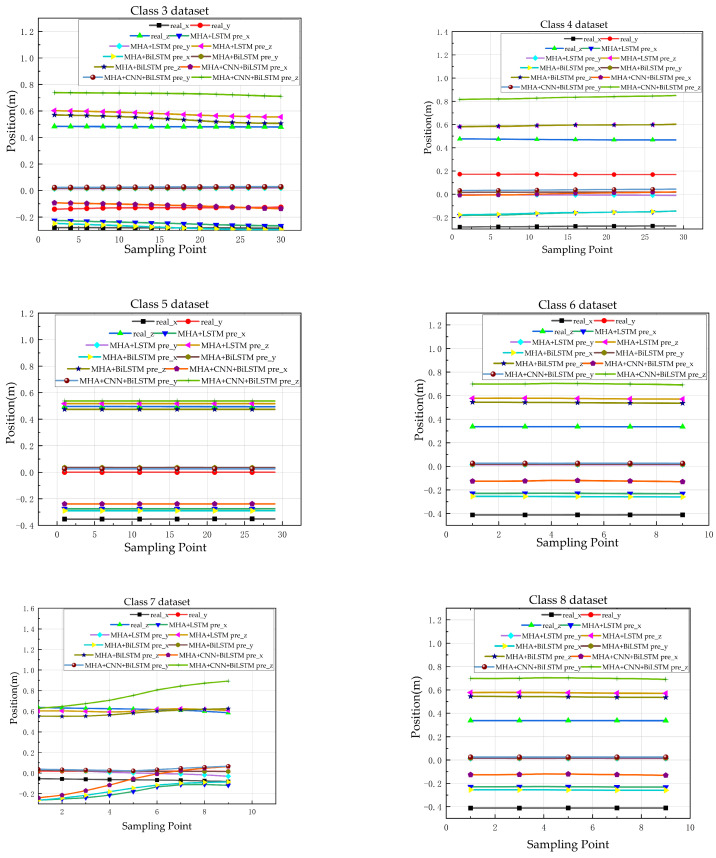
Results of the three predictive models X, Y, and Z on eight test set actions.

Figure 12 presents the prediction results of three algorithms on eight different datasets. Overall, the three algorithms perform well on Dataset 1 and Dataset 2, but MHA + CNN + BiLSTM exhibits the largest prediction error along the *X*-axis for Dataset 2. When predicting Dataset 3, the precision of MHA + CNN + BiLSTM is the lowest for the *Z*-axis, followed by MHA + LSTM. Due to the influence of temporal differences in Dataset 4, the average errors along the *Z*-axis for MHA + CNN + BiLSTM and MHA + LSTM are 0.4 m and 0.2 m, respectively, both of which are greater than that of MHA + BiLSTM. On Dataset 5, all three algorithms exhibited suboptimal performance, with MHA + CNN + BiLSTM having the lowest precision and an average error of 0.18 m, while MHA + LSTM and MHA + BiLSTM had average errors of 0.05 m. For Dataset 6 and Dataset 8, influenced by individual differences, MHA + BiLSTM demonstrated the highest predictive accuracy. Datasets 6 and 8 are influenced by individual differences, with MHA + BiLSTM demonstrating the highest predictive accuracy. In Dataset 7, both MHA + BiLSTM and MHA + LSTM exhibit relatively high prediction accuracy, while MHA + CNN + BiLSTM has the largest prediction error. To verify the performance of the MHA Bi-LSTM method in the real-time prediction of differential trajectories, we predicted human hip sitting trajectories using MHA Bi-LSTM. We performed online predictions of 15 sample points for three different motion trajectories and quantitatively analyzed them using the mean error, RMSE, and MAE. The detailed analysis results are given in Table 2.

The calculation equation is given in the following Equations (16) and (17):

(16) $$\mathrm{RMSE}=\sqrt{\frac{1}{n}\overset{n}{\underset{i=1}{\Sigma }}{\left({\hat{y}}_{i}-y_{i}\right)}^{2}}$$

(17) $$\mathrm{MAE}=\frac{1}{n}\overset{n}{\underset{i=1}{\Sigma }}\left({\hat{y}}_{i}-y_{i}\right)$$

where *n* is the sample size, *ŷ* the predicted value, and *y* is the true value.

The following paper conducted experiments on the MHA Bi-LSTM neural network prediction model to make real-time online predictions. According to the results in Table 2, the highest mean prediction error was 0.043 m, and the lowest was 0.010 m. Notably, the accuracy of predicting the position of Y was lower than the accuracy of predicting other types of motion in the down-sit to the left offset test set. Table 2 analyzed these three types of motion using three evaluation models: mean error, RMSE, and MAE.

### 4.3. Validation of Transfer Assist Using the ECR

**Figure 13:**
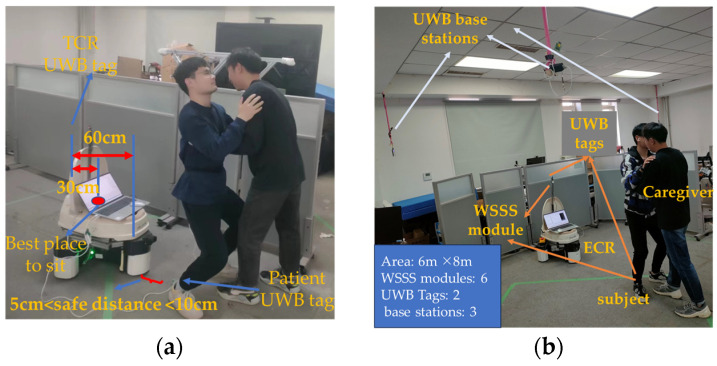
Experimental environment and location of ECRs with regards to subjects. (**a**) ECR in consideration to subject location. (**b**) Experimental environment.

In the experiments, the ECR’s UWB tag was placed near the rear of the ECR, which is 60 cm from the very front of the ECR, the human body’s UWB tag was placed at the ankle, and the safe distance between the ECR and the human body was set at 5 cm to 10 cm for patient safety. When the robot obtains the human toilet point in advance, it first determines whether the posture is consistent, then the ECR moves towards the target point and finally fine-tunes the angle to provide better service. We judge the distance from the ECR to the buttocks during the ECR traveling process and then adjust the ECR to move along the *X*-axis or *Y*-axis until it reaches the toilet point. A UWB tag and a WSSS module are placed on the ECR and the right foot of the human body for localization and determination of the Yaw. Figure 13a,b provides the details of the experimental setting and the location of the ECR with regard to the subjects.

The purposes of the experiments in this subsection are to validate the prediction performance of MHA Bi-LSTM in a real-time environment for different individuals and differences in sitting times with assistance.

**Figure 14:**
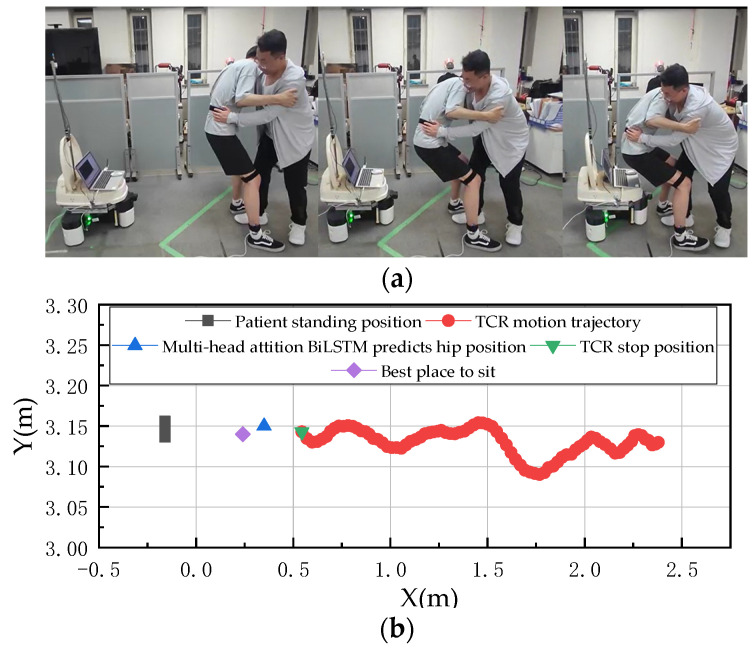
Human Transfer Care Experiment. (**a**) Details of the human sitting experiment without deflection. (**b**) UWB real-time positioning.

The specific procedure for the first set of experiments was as follows, with the process and details of this set of experiments shown in Figure 14a, and the traveling path of the UWB tracking ECR and the position of each point shown in Figure 14b:

The first group of experiments is set up without deflection when the human body sits down. From Figure 14a, we can see that the position of the right foot of the human body is measured by UWB as A1 (−0.130, 3.150), the position of the ECR is measured as C1 (0.540, 3.140), and the MHA Bi-LSTM predicts that the coordinates of the point where the human body sits down are B1 (0.350, −0.011), and the point B1 is translated into the world coordinate system as B11 (0.220, 3.161). From Figure 14, it can be seen that the best down-sitting point is 30 cm from point C1, namely, D1 (0.240, 3.140). An analysis of points B1 and D1 shows an error of 0.020 m in the *X*-axis direction and an error of 0.021 m in the *Y*-axis direction, and the error in the *X*-axis direction can be reduced by adjusting the safety distance. The error in the *Y*-axis can be experimentally accepted.

**Figure 15:**
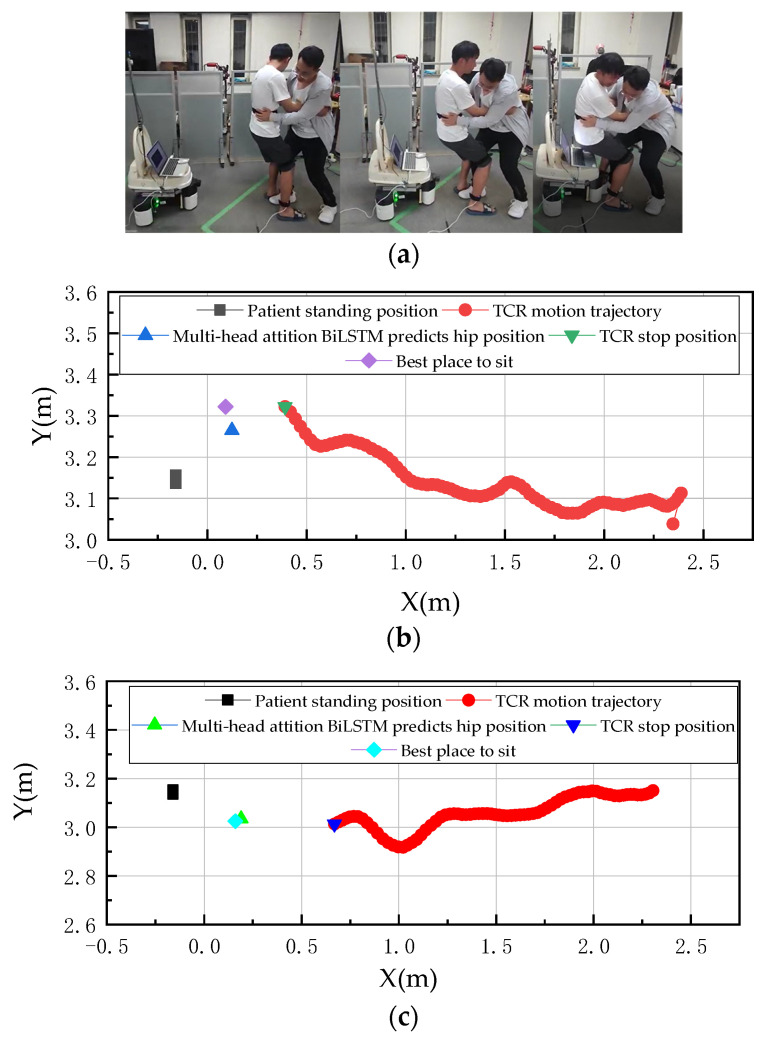
Down-sit right offset multiplier care experiment. (**a**) Down-sit right bias experiment procedure. (**b**) Scenario 1. (**c**) Scenario 2.

The specific experimental procedure of the second group is as follows: The second participant was assisted to sit with their hips moving to the right. Figure 15a presents the relevant details of the experiment, while Figure 15b provides the UWB tracking and human positioning data related to the ECR movement path. The second participant was assisted to sit with their hips moving to the left, as shown in Figure 15c, which presents the corresponding details of the experiment.

In the second set of experiments, the right foot position A2 (−0.160, 3.140) and the ECR position C2 (0.468, 3.304) were measured via UWB, and the MHA Bi-LSTM prediction was used to obtain the human body sitting down point B2 (−0.301, 0.125), which was converted to the world coordinate system B22 (0.141, 3.275), and the best sitting down point was D2 (0.168, 3.304). The analysis of B22 and D2 showed that there was an error of 0.027 m in the X direction and 0.029 m in the Y direction.

Figure 15c provides experimental details for Experiment 3. In experiment 3 we conducted the experiments with the same experimenters at different times, and Figure 15c provides experimental data on the UWB tracking and human localization of ECR movement paths in this set of experiments. In the third group of experiments, the UWB prediction was used to obtain the sitting point A3 (−0.160, 3.140), the ECR position C3 (0.476, 3.011), the MHA Bi-LSTM prediction of the human sitting point B3 (−0.325, 0.153), the conversion of B3 to the world coordinate system B33 (0.165, 2.987), and the best toilet sitting point D3 (0.176, 3.011). By analyzing the B33 and D3 points, we can see that the error in the *X*-axis direction is 0.01 m, and the error in the *Y*-axis direction is 0.024 m.

In summary, the proposed prediction model of MHA Bi-LSTM demonstrates good predictive performance and robustness when applied to transfer actions involving individual differences and temporal variations.

## 5. Conclusions

In this study, we captured lower limb motion data using wearable sensors and developed a lightweight 3D human body model to reconstruct human motion. The 3D model was used to obtain the three-dimensional trajectory of the hips. Subsequently, we combined the MHA and Bi-LSTM models to predict individualized transfer movements, considering the variations among individuals. We also compared our proposed method with two other time-series-based neural network models to validate its reliability. The preliminary experimental results demonstrate that the introduced MHA Bi-LSTM model achieves higher accuracy and requires less computation time in predicting the wearable-sensor-based toileting actions of the human body. In future work, we will provide more test results of the MHA Bi-LSTM model on transfer movements with different characteristics, and continue to investigate other assessment metrics and improvement strategies for ECRs, to assist caregivers in reducing caregiver regression and improving patients’ willingness to toilet.

## Figures and Tables

**Table 1:** Results of the multi-point calibration method.

Model		Calibrated Position				Measurement Position			
		A1	B1	C1	D1	A2	B2	C2	D2
Subject normal	X	0.33 m	0.26 m	0.16 m	0.10 m	0.4 m	0.25 m	0.13 m	0.12 m
	Y	0 m	0 m	0 m	0 m	0.01 m	0.02 m	0.01 m	0.03 m
	Z	0.46 m	0.49 m	0.33 m	0.43 m	0.50 m	0.49 m	0.34 m	0.44 m
Subject left	X	0.23 m	0.30 m	0.13 m	0.08 m	0.12 m	0.27 m	0.06 m	0.1 m
	Y	0.16 m	0.22 m	0.12 m	0.24 m	0.17 m	0.23 m	0.14 m	0.27 m
	Z	0.45 m	0.45 m	0.33 m	0.45 m	0.49 m	0.51 m	0.30 m	0.49 m
Subject right	X	0.26 m	0.28 m	0.14 m	0.05 m	0.24 m	0.28 m	0.10 m	0.07 m
	Y	0.18 m	0.24 m	0.10 m	0.11 m	0.21 m	0.22 m	0.09 m	0.11 m
	Z	0.48 m	0.49 m	0.33 m	0.46 m	0.51 m	0.5 m	0.30 m	0.48 m

**Table 2:** Mean, RMSE, and MAE of the prediction errors.

Model		Error		
		Mean	RMSE	MAE
No offset	X	0.038 m	0.021 m	0.041 m
	Y	0.012 m		
	Z	0.010 m		
Offset to the right	X	0.021 m	0.022 m	0.022 m
	Y	0.024 m		
	Z	0.022 m		
Offset to the left	X	0.043 m	0.041 m	0.034 m
	Y	0.032 m		
	Z	0.023 m		

## Data Availability

No new data were created or analyzed in this study. Data sharing is not applicable to this article.
